# Small cell lung cancer: model of circulating tumor cell tumorospheres in chemoresistance

**DOI:** 10.1038/s41598-017-05562-z

**Published:** 2017-07-13

**Authors:** Lukas Klameth, Barbara Rath, Maximilian Hochmaier, Doris Moser, Marlene Redl, Felicitas Mungenast, Katharina Gelles, Ernst Ulsperger, Robert Zeillinger, Gerhard Hamilton

**Affiliations:** 1 Department of Pathophysiology and Allergy Research, Medical University of Vienna, Vienna, Austria; 2 Deparment of Surgery, Medical University of Vienna, Vienna, Austria; 3 Respiratory Oncology Unit, Otto-Wagner Hospital, Vienna, Austria; 4 Department of Cranio-Maxillofacial and Oral Surgery, Medical University of Vienna, Vienna, Austria; 5 Hospital Horn, Horn, Austria; 6 Department of Gynecology, Medical University of Vienna, Vienna, Austria

## Abstract

Small cell lung cancer (SCLC) represents 15% of lung cancers and is characterized by early dissemination, development of chemoresistance and a poor prognosis. A host of diverse drugs failed invariably and its mechanisms of global chemoresistance have not been characterized so far. SCLC represents the prototype of an aggressive and highly metastatic tumor which is ultimately refractory to any treatment. High numbers of circulating tumor cells (CTCs) allowed us to establish 5 CTC cell lines (BHGc7, 10, 16, 26 and UHGc5) from patients with recurrent SCLC. These cell lines exhibit the typical SCLC markers and CTCs of all patients developed spontaneously large multicellular aggregates, termed tumorospheres. Ki67 and carbonic anhydrase 9 (CAIX) staining of tumorosphere sections revealed quiescent and hypoxic cells, respectively. Accordingly, comparison of the chemosensitivity of CTC single cell suspensions with tumorospheres demonstrated increased resistance of the clusters against chemotherapeutics commonly used for treatment of SCLC. Therefore, global chemoresistance of relapsing SCLC seems to rely on formation of large tumorospheres which reveal limited accessibility, lower growth fraction and hypoxic conditions. Since similar tumor spheroids were found in other tumor types, SCLC seems to represent a unique tumor model to study the association of CTCs, metastasis and drug resistance.

## Introduction

Small cell lung cancer (SCLC) is an aggressive neuroendocrine lung tumor representing 15% of lung cancers which is found disseminated in most patients at the time of first presentation^1, 2^. The great majority of patients consist of smokers with heavy tobacco consumption for decades^3^. Genetically, SCLC is characterized by inactivation of tumor suppressors p53 and retinoblastoma gene (Rb1) and additional mutations or increased expression of a wide range of different drivers for small subpopulations of patients^1, 4^. Current standard care for extended stage disease (ED-SCLC) or metastatic SCLC is platinum-based chemotherapy and thoracic/prophylactic cranial irradiation^5, 6^. Despite very high initial response rates, tumors recur rapidly within 1–2 years comprising local relapse or metastasis to liver, brain, bone and other secondary sites. Second-line therapy employs the single approved chemotherapeutic topotecan or anthracycline-based therapy as alternative but yields poor responses of short duration^5, 6^. A host of studies performed over the last decades evaluating all kinds of conventional chemotherapeutics, novel drugs, targeted and antiangiogenic agents failed to provide a survival benefit in SCLC over standard chemotherapy^5–7^. The poor prognosis of SCLC, with a typical overall 5-year survival of 5–10% which has not changed considerably for the last decades, requires new modalities of treatment guided by a better understanding of the underlying biology of tumor spread and development of drug resistance. Tumor relapse and spread in SCLC seems to be related to the extraordinary high counts of circulating tumor cells (CTCs) which exceed CTC numbers in other malignancies by orders of magnitude^8, 9^. Part of these CTCs were suggested to exhibit a general chemoresistance and to be able to induce secondary lesions. Most CTCs perish in the peripheral circulation and only a small subpopulation has tumor-initiating properties but these actual tumorigenic cells are not identifiable among the heterogeneous cancer cells populations^10^. In an attempt to isolate and expand the SCLC CTCs *in vitro*, we were successful in establishing 5 CTC cell lines so far which grow continuously in tissue culture^11, 12^. These SCLC CTC lines derived from different patients exhibit similar expression of proteins, cytokines and various receptors, indicating their important role in metastatic SCLC. A cell biological profile of such CTCs stemming from relapsed SCLC patients could now be investigated to find putative mechanisms of chemoresistance for the first time. SCLC seems to represent the prototype of a tumor capable of aggressive growth, rapid generation of CTCs, acquisition of a universal chemoradioresistant phenotype and low survival. Access to the SCLC CTC lines offers a singular opportunity to study the interrelation of CTCs, metastasis and development of resistance which may apply to other tumor types which display these processes in a more protracted manner as well.

## Results

### SCLC marker expression in CTC cell lines

**Figure 1:**
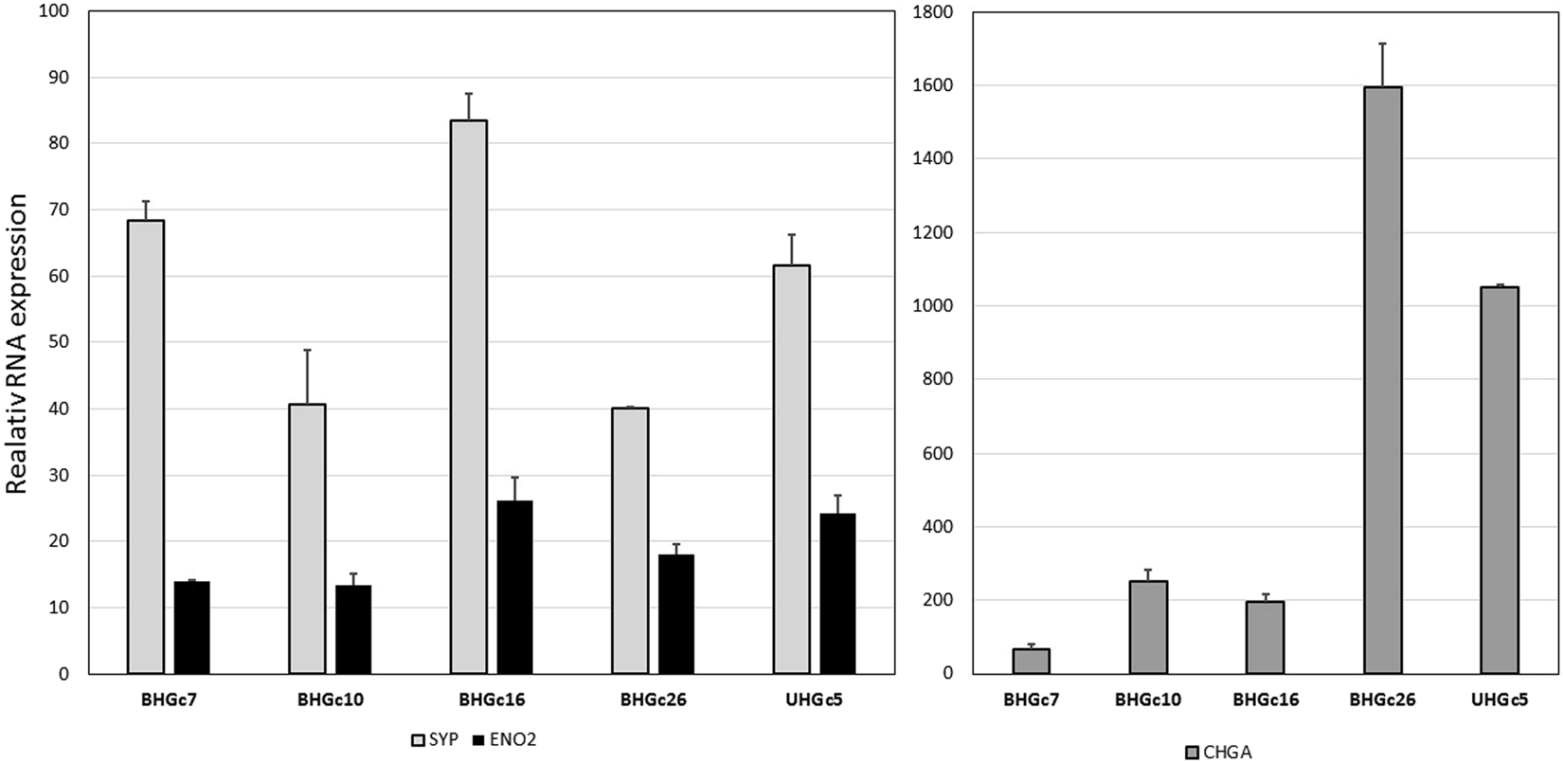
RT-qPCR analysis of the expression of SCLC markers of SCLC CTC lines. Expression of the markers is shown as mean values ± SD relatively to normal lung tissue.

Expression of typical SCLC markers synaptophysin (SYP), enolase-2 (ENO2) and chromogranin A (CHGA) of the CTC lines BHGc7, 10, 16, 26 and UHGc5 were tested in RT-qPCR relatively to RNA from normal lung tissue (Fig. 1). All SCLC CTC lines were found to show marked expression of all 3 markers, corresponding to their SCLC origin. Whereas expression of SYP and ENO2 was similar in the five CTC lines, expression of CHGA varied widely and is normally correlated with the content of intracellular secretory vesicles. An additional marker, namely CD56/NCAM, is shown in immunohistochemistry for the CTC tumorospheres below.

### Light and scanning electron microscopy (SEM) of CTC tumorospheres

**Figure 2:**
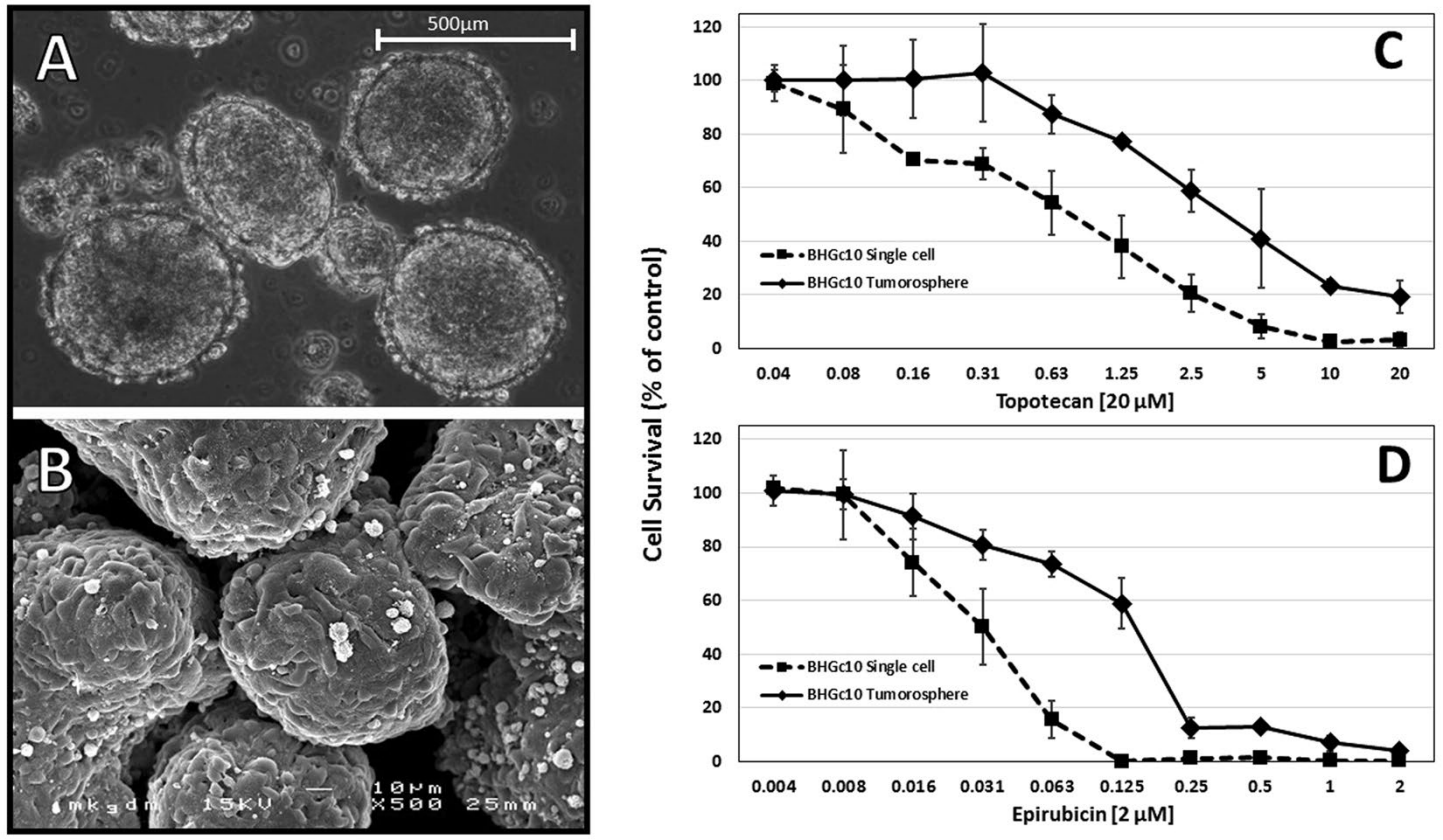
Light and scanning electron microscopy and chemosensitivity of CTC tumorospheres. (**A** and **B**) show light and SEM images of BHGc26 tumorospheres, respectively; (**C** and **D**) a comparison of the chemosensitivities to topotecan and epirubicin of BHGc10 single cells and tumorospheres in MTT assays, respectively (For the cytotoxicity assays, measurements are shown as mean values ± SD).

Light microscopy of the CTC cultures revealed large multicellular clusters, termed tumorospheres, which grow to sizes of 1–2 mm in diameter and show a dark core region for larger aggregates (Fig. 2A). In contrast to other tumor spheroids described, these tumorospheres develop in all 5 CTC lines spontaneously in regular tissue culture medium without the need to inhibit cellular adherence to substrates. In SEM, tumorospheres appear as multicellular aggregates with outer cells forming a densly closed spheroid surface (Fig. 2B).

### Cytotoxicity tests of SCLC CTCs as single cell and tumorospheres

Equivalent CTC cell numbers either in form of single cell suspensions or as tumorospheres were exposed to topotecan and epirubicin, respectively, which are commonly used as second-line chemotherapeutics in relapsed SCLC. Tumorospheres used in these cytotoxicity assays exhibit diameters of up to 300 µm. Dose-response curves for these two drugs and the BHGc10 CTC cell line showed a significant decrease of the chemosensitivities of tumorospheres to both drugs in comparison to single cell cultures (Fig. 2C,D).

**Figure 3:**
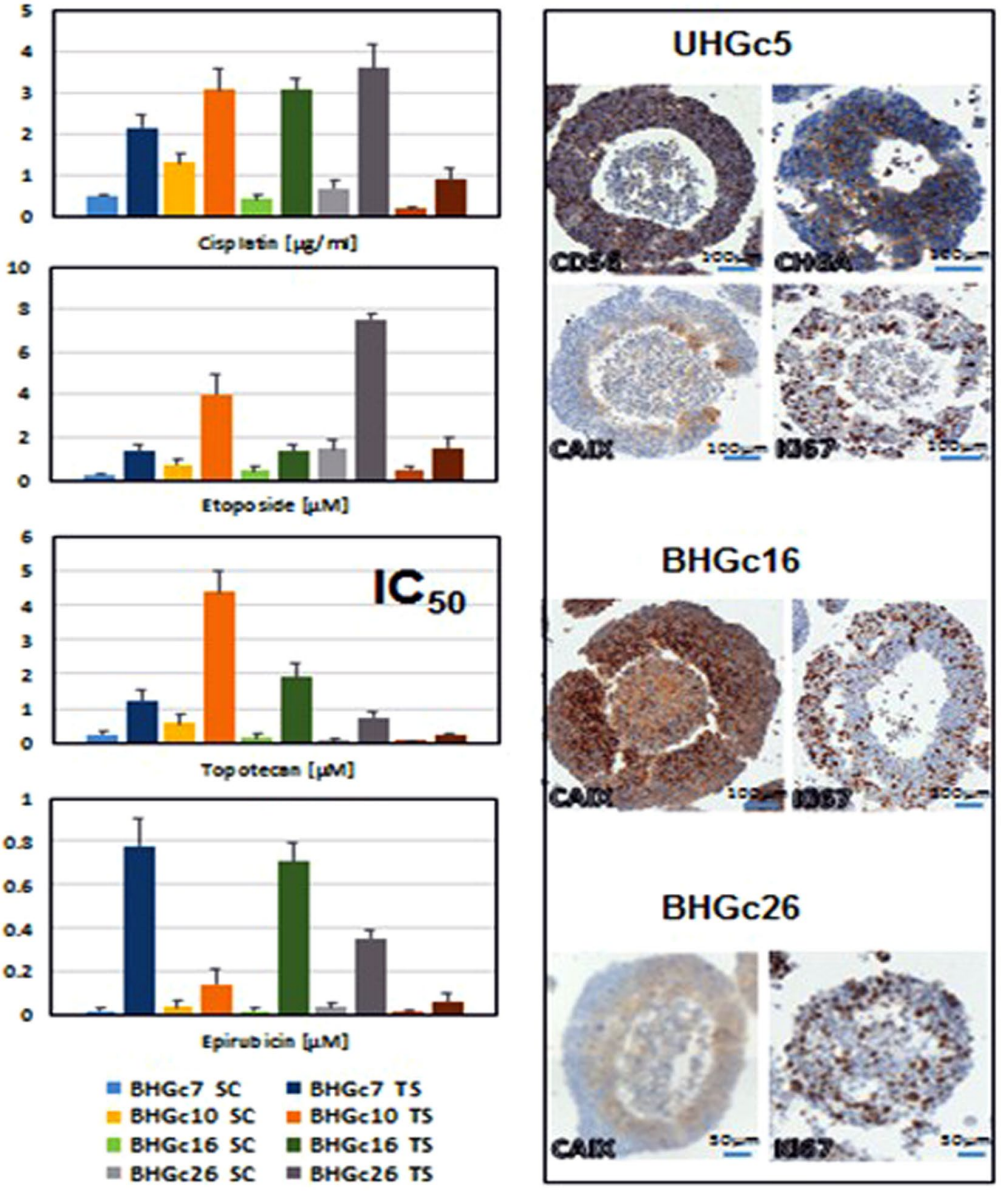
Chemosensitivity of the CTC SCLC lines (IC_50_, mean ± SD) and immunohistochemistry of sections of tumorospheres. All differences between single cells (SC) and tumorospheres (TS) are statistically significant. Immunohistochemical staining of sections of UHGc5, BHGc16 and BHGc26 CTCs was performed using antibodies directed to CD56, CHGA, CAIX and Ki67, respectively.

These cytotoxicity tests were performed for all 5 SCLC CTC cell lines and extended to include cisplatin and etoposide which constitute the most common administered first-line cytotoxic drugs for SCLC patients in combination. The resulting IC_50_ values are shown in Fig. 3, left side. As single cell suspensions, all CTC lines are chemosensitive to the drugs tested, except for BHGc10 and cisplatin. BHGc10 was established from a patient refractory to primary chemotherapy comprising the platinum/etoposide combination. Again, all CTC lines in form of tumorospheres exhibit significantly higher chemoresistance to all 4 chemotherapeutics in comparison to the same cells employed as single cell suspensions. The mean increase in chemoresistance was 4.8 ± 1.8 fold for cisplatin, 4.6 ± 1.3 fold for etoposide, 9.0 ± 3.2 fold for topotecan for all CTCs, and approximately 50 fold for BHGc7 and 16 and 6.8 ± 2.5 fold for BHGc10 and 26 and UHGc5 for epirubicin, respectively.

For further characterization, CTC tumorospheres were fixed, embedded in paraffin and sections prepared for immunohistochemistry. The right side of Fig. 3. shows sections of UHGc5 tumorospheres stained for the SCLC markers CD56/NCAM and CHGA as well as carbonic anhydrase IX (CAIX) and the proliferation marker Ki67. In addition, BHGc16 and 26 tumorospheres with CAIX and Ki67 staining are depicted. All tumorospheres of larger sizes reveal a necrotic core which has been visible as dark central region in light microscopy. In correspondence with the RT-qPCR results, all tumorospheres stain positively with antibodies to the SCLC markers CHGA and CD56/NCAM. The CAIX expression is either confined to regions close to the core, as for UHGC5 and BHGc26, or reveals a more extended distribution as in BHGc16 aggregates. The frequency of cells in the proliferative phase, as indicated by Ki67 staining, is higher in the peripheral regions of the tumorospheres and lower or absent in the cores. Similar results were found for tumorospheres originating from the CTC cell lines BHGc7 and 10. At very large sizes exceeding 1.5–2 mm the tumorospheres tend to disintegrate in tissue culture (data not shown).

### Growth fraction of single cells and tumorospheres of CTC lines

**Figure 4:**
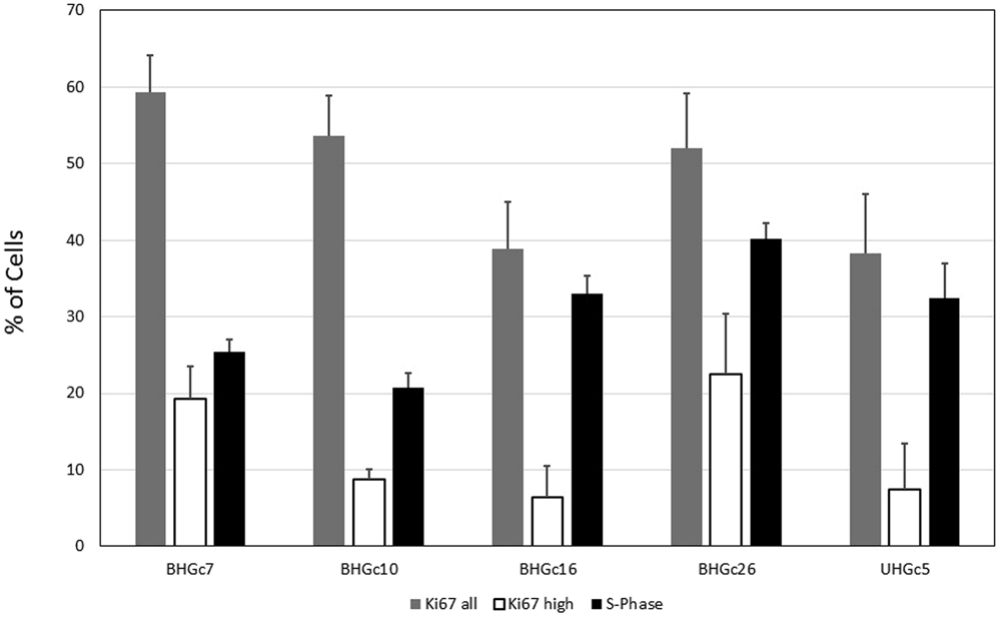
Growth fractions of SCLC CTCs as determined by Ki67 and PI DNA staining. Percentages of Ki67 and S-phase cells are presented as mean ± SD. All differences between the S-phase fractions as determined by Ki67^high^ and the PI flow cytometric method are statistically significant.

Growth fractions of CTCs were measured either by quantitiative image analysis of Ki67 expression of the embedded tumorosphere sections or propidium iodide (PI) DNA staining of the cells from single cell suspension cultures (Fig. 4). Of the results from PI cell cycle analysis, the S-phase fraction is presented for the CTC lines. The antibody directed to Ki67 stains all cells, except those in the G0/1 resting phase, with variably intensity but actual proliferating G2M phase cells are characterized by highest expression of this antigen. The fraction of Ki67^high^ cells is significantly lower compared to the S-phase fraction, as measured by the PI assay of single cell suspensions, suggesting a lower growth fraction of CTC SCLC cells which are part of the tumorospheres.

### Soluble CAIX expression by CTC lines single cells cultures and tumorospheres

**Figure 5:**
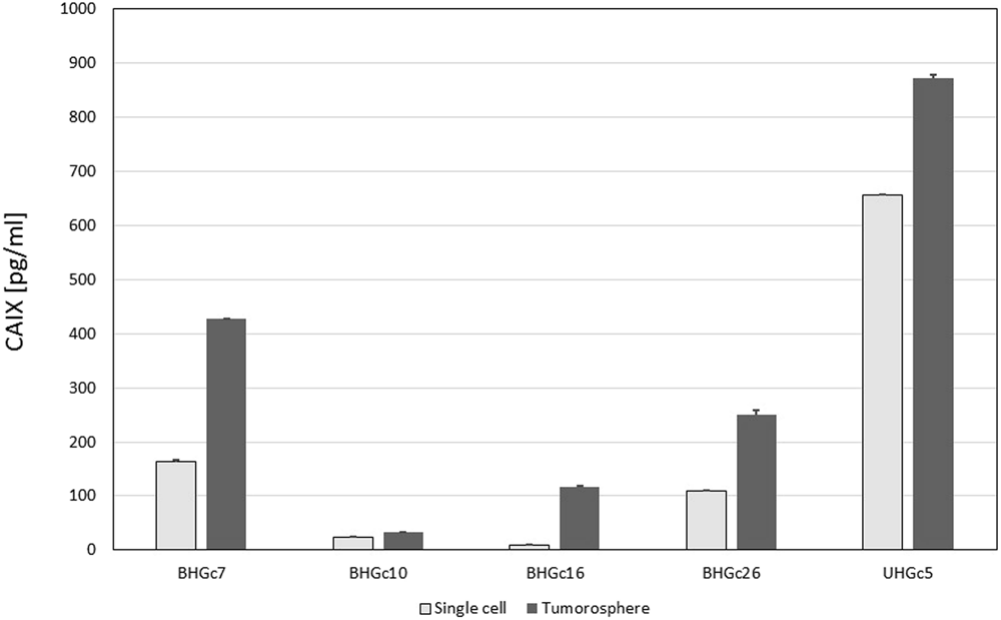
Expression of sCAIX in supernatants of single cell SCLC CTCs and tumorospheres. CTC cell lines were cultivated for one week and sCAIX was determined in supernatants using an ELISA assay. Values are shown as mean ± SD. All differences between single cells and tumorospheres are statistically significant.

CAIX is expressed either as membrane-bound enzyme and or as an extracellular soluble fragment (sCAIX) lacking the transmembrane part but retaining the active enzymatic site. ELISA measurements of sCAIX demonstrated low levels of this antigen in single cell suspensions of the CTC lines, with exception of UHGc5 which released significant amounts of this antigen under normoxic conditions (Fig. 5). Tumorospheres showed a significant higher release of sCAIX compared to the single cell cultures of the CTCs, indicating more hypoxic conditions in these multicellular aggregates. HIF1α protein expression was tested in Western Blots but was found in BHGc7 clusters at low levels (data not shown), possibly indicating intermittent oxygenation of the clusters which results in transient expression of this transcription factor but continuing expression of CAIX which possesses a hypoxia-responsive element (HRE) in its promotor.

## Discussion

Extended disease SCLC is characterized by early failure of chemoradiotherapy and low activity of all chemotherapeutics tried in the second-line setting^1, 2^. So far, it is not clear how this kind of broad chemoresistance develops following the excellent responses in most patients during first-line therapy^4^. Apparently, a few primary or disseminated tumor cells survive the first cycles of chemotherapy and generate relapses within a period of approximately one year. This would fit the cancer stem cell (CSC) model which describes tumor cell recurrence as expansion of a small subpopulation of pluripotent and highly resistant cancer cells which survive initial chemotherapy^13^. SCLC exhibits extremely high counts of CTCs which are clearly involved in tumor spread and relapse and may comprise a subpopulation of CSCs^9^. However, we showed that the SCLC CTC cell lines are highly sensitive to topotecan and epirubicin as single cells in *in vitro* cytotoxicity assays and this phenotype is not compatible with the characteristics of drug-resistant CSCs. Furthermore, we tested the SCLC CTC lines for expression of typical CSC markers, such as CD133, ABCG2 and sensitivity to salinomycin, but obtained negative results except for the single expression of CD133 in a CTC line established from a patient with primary resistance to cisplatin (manuscript submitted)^14^. Therefore, SCLC CTCs must exploit mechanisms of survival different from common cellular alterations and pathways resulting in chemoresistance, such as increased drug efflux, inactivation or mutation of targets, increased repair and others. Additionally, individual drug resistance mechanisms for a wide range of unrelated chemotherapeutics have not been demonstrated in SCLC cells and attempts to reverse suppression of apoptosis were not successful^15, 16^.

All SCLC CTC cultures showed spontaneous formation of large multicellular aggregates which grow to diameters of 1–2 mm, designated tumorospheres, under regular cell culture conditions^11, 12^. Such spheroidal and organized cell arrangements are not observed in the SCLC cell lines, which grow as loosely connected and irregular cell clusters^12^. In most systems, tumor spheroids are only formed in response to culture conditions which prevent attachment of the cells, such as hanging drop cultures or use of low-adherence substrates and other methods^17, 18^. Tumorospheres grown to large sizes of up to several 100 μm are expected to show a lack of nutrients and oxygen as well as an accumulation of waste products in their interior^19, 20^. In comparison to two-dimensional (2D) cell cultures, 3D cultures or spheroids exhibit increased resistance to chemotherapeutics and irradiation due to presence of quiescent cells, cell contact-mediated effects and lack of generation of oxygen radicals^21–25^. Cancer cells grown *in vitro* as spheroids represent the chemoresistance phenotype of native solid tumors exactly and display pathways of resistance linked to hypoxia, alterated chromatin structure, impairment of apoptosis, cell cycle alterations and decreased drug perfusion^26^. All 5 SCLC CTC lines express typical neuroendocrine markers and form tumorospheres with enclosed surfaces and hypoxic/necrotic cores. Ki67^high^ expressing cells are enriched in the peripheral region and quiescent cells near the core and expression of CAIX in the inner regions indicate at least transient hypoxia^27, 28^. Expression of HIF1α may be short-lived but turnover of CAIX is slowly. Correspondingly, the release of sCAIX is higher in supernatants of tumorospheres compared to single cell suspensions^29^. Comparative cytotoxicity tests employing 4 common drugs used in the therapy of SCLC reveal significantly increased chemoresistance of all tumorospheres which may account for the observed clinical refractoriness of relapsed patients. This barrier function of tumorospheres and decreased sensitivity of quiescent cells can explain the broad-range drug resistance in relapsed SCLC as hindrance to reach an efficient level of the agents at the target site and to kill insensitive cells altered in a multicellular assembly^19, 20^.

*In vivo* tumor growth is characterized by the development of hypoxic and necrotic areas caused by variable supply of oxygen and shifting supply of nutrients. The expression of numerous genes differs between cells growing as MCTS as compared to monolayer cultures. Cell lines from tumor and normal tissues kept as monolayers display a downregulation of the number and levels of differentially expressed genes by up to 70%^30^. Increasing colorectal cancer spheroid sizes are characterized by initial normoxia, subsequent hypoxia, and eventual hypoxia plus necrosis, respectively^26^. MCTSs which most closely resemble gene expression profiles of *in vivo* growing tumors and exhibit the highest chemoresistance are present in cell lines of most solid cancers and pluripotent stem cell cultures^19, 20, 31^. In ovarian cancer patients, spheroids can also form by budding directly as clusters from cell monolayers and were found to acquire progressive resistance to the chemotherapeutics paclitaxel and cisplatin^32^. This type of chemoresistance could be completely reversed by dissociation of the spheroids. Quiescent colon adenocarcinoma tumor spheroids showed decreased expression of the proliferation marker Ki67 and increased expression of the quiescence marker p27(Kip1) compared to proliferating spheroids^25^. The quiescence was completely reversible demonstrating that the quiescent cancer cells retained the ability to proliferate. The cytotoxicity of doxorubicin and cisplatin was significantly reduced in spheroids containing quiescent cells and, furthermore, 5-FU and vinblastine showed no cytotoxicity. Contrary to 2D cultures, no significant loss of viability was observed in 3D systems of endometrial cancer in response to doxorubicin and cisplatin^33^.

**Figure 6:**
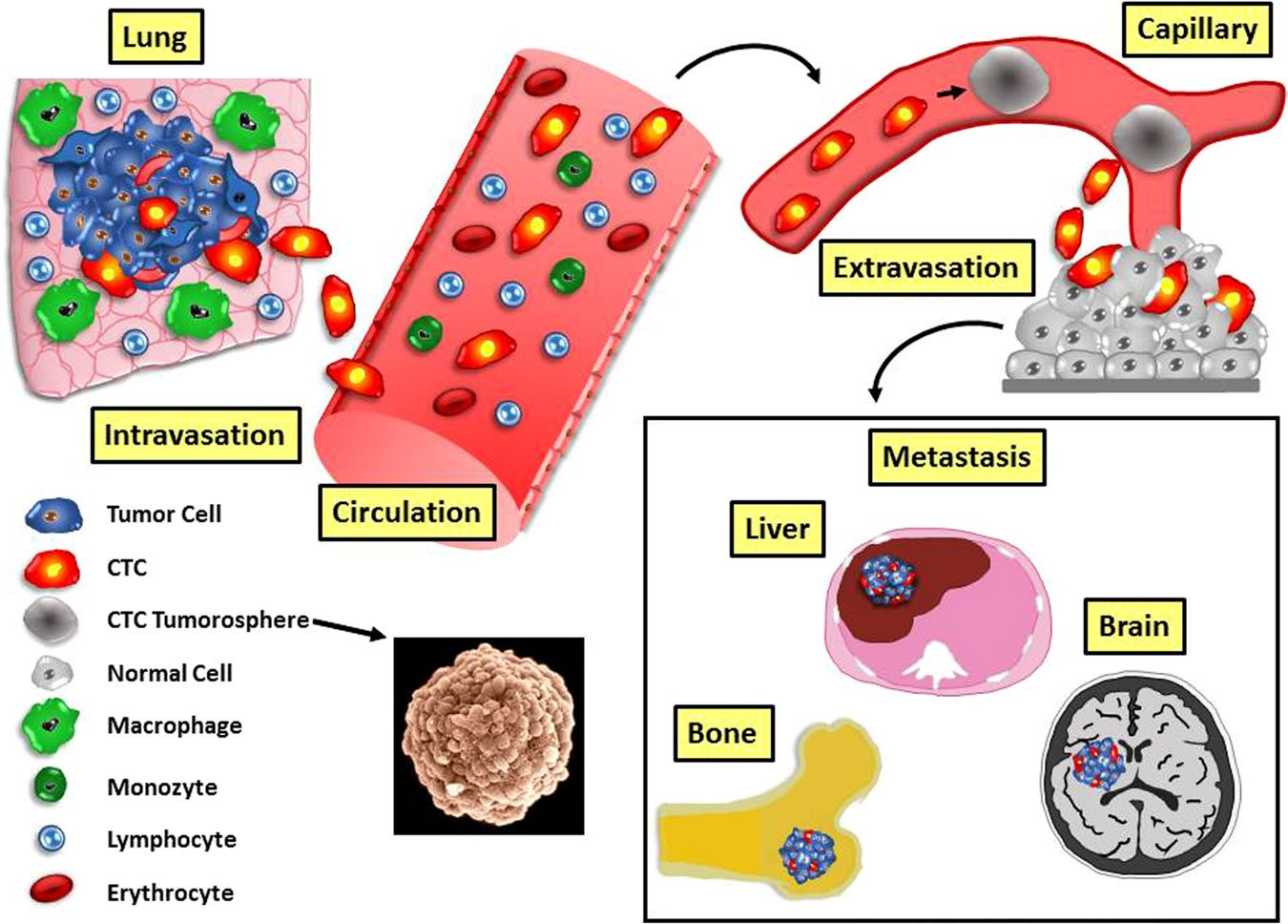
Scheme of the model of tumor dissemination including tumorospheres. SCLC originates in the lung and CTCs are shed from the tumor and intravasate into the circulation. CTCs or small clusters of CTCs may become trapped in small capillaries of distal sites and grow into large tumorospheres which are resistant to radiochemotherapy. Eventually tumor cells derived from tumorospheres or the whole spheroid extravasate and grow aggressively into secondary lesions preferentially in liver, bone and brain. Tumorospheres may still be present as part of the metastases and this model may likewise apply to related tumors, such as glioblastoma and neuroectodermal tumors which show a similar clinical course and dismal prognosis.

Despite high investments to develop new cancer therapeutics, about 90% of drugs finally tested in clinical trials fail^34, 35^. Spheroids with sizes of 250–750 µm are detectable in cancer patients and, correspondingly, traditional testing methods are increasingly being complemented by 3D models of human tumors. However, the underlying assumption, that multicellular spheroids formed through enforced detachment of cells from substrates by techniques such as hanging drops, low adherence culture plates or other means, are largely identical to spontaneously originating tumorospheres, has not been validated. Although artificially prepared spheroids may be suitable to test some aspects of resistance to drugs, targeting the formation of clusters or attempts to disintegrate spheroids may depend on the specific factors contributing to spontaneous assembly of the aggregates under natural conditions^36, 37^. For relapsed SCLC, the failure of a host of chemotherapeutics to improve therapy and survival seems to be linked to the formation of tumorospheres, which provide higher chemoresistance against chemically diverse drugs indiscriminately (Fig. 6). Spheroid cultures were demonstrated to differentially express genes involved in extracellular matrix organization and cell adhesion but not in cellular mechanisms of drug resistance and isolated cluster cells are chemosensitive^30, 32^. In conclusion, chemotherapy responsiveness of CTCs from relapsing SCLC patients is likely to be determined by the protein interactome involved in cell aggregation which is difficult to deduce from transcriptomics data. Universal chemoresistance due to formation of large clusters may not be limited to SCLC but occur in other related tumors, such as glioblastoma and neuroectodermal tumors, as well. Furthermore, individual metastatic cancer cells in peritoneal fluid derived from the ovary and gastrointestinal tract can form multicellular spheroids which are resistant to anoikis, apoptosis, and chemotherapeutics^38, 39^. Demonstration of spheroid-mediated chemoresistance in SCLC CTCs points to completely new ways to tackle the poor survival rates of this aggressive malignancy, although effective clinical strategies to overcome this type of physical barrier are currently not available. The SCLC CTC lines supply spontaneously formed spheroids which seem to provide *in vitro* equivalents of actual *in vivo* multicellular structures and can be used to study metastasis via CTCs, drug resistance and advanced therapeutic modalities for attacking 3D-tumor structures. Tumorosphere-related drug resistance emulates the clinical picture of the putative CSC-associated resistance which has not translated to clinical successful treatments so far^40^.

## Materials and Methods

### CTC lines and cell culture

SCLC CTC cell lines BHGc7, 10, 16, 26 and UHGc5 were established previously in our lab from patients with ED-SCLC^11^. Blood collection and generation of cell lines was done after receiving informed consent according to the Ethics Approval 366/2003 of the Ethics Committee of the Medical University of Vienna, Vienna, Austria. Cells were cultivated in RPMI-160 medium (Biochrome, Berlin, Germany) supplemented with 10% fetal bovine serum (Biochrome) and antibiotics (Sigma-Aldrich, St.Louis, MO, USA) under tissue culture conditions and split following detachment with cell scrapers (TPP, Trasadingen, Switzerland).

### Light and electron microscopy

#### Embedding of the tumorospheres

Paraformaldehyde-fixed CTC clusters were separated from adherent cells by washing with PBS and transferred into a fresh reaction tube. Clusters were centrifuged at 400 g and the supernatant was removed. Subsequently, clusters were washed with 10 ml distilled water for 30 minutes under frequent mixing. Clusters were transferred into a 1.5 ml reaction tube, centrifuged and water was replaced by 70% ethanol for one hour under frequent mixing at room temperature. This step was repeated with 85%, 96% ethanol and isopropanol, each for one hour. Isopropanol was removed as completely as possible and lower melting paraffin (52–54 °C) was added. For the first 15 minutes, the tube was inverted in regular intervals and then incubated overnight at 60 °C. The next morning, the paraffin was discarded as completely as possible and higher melting paraffin (56–58 °C) was added. Again, the tube was inverted in regular intervals within the first 15 minutes and then incubated overnight at 60 °C. Afterwards, the tube was cooled at room temperature till the paraffin solidified. The tip of the tube, which contains settled CTC clusters, was cut off with a scalpel and the paraffin tip was embedded in a paraffin block. 4 µM thick sections were cut with the Microtom HM 355 S (Thermo Fisher, Waltham, MA, USA) and dried overnight at 37 °C.

#### Immunohistochemistry of the tumorosphere sections

Cluster sections were deparaffinized at 60 °C for 25 minutes. Afterwards, rehydration took place by incubation for 3 × 10 minutes with xylol and five minute incubations with absolute, 96%, 70%, 50%, 30% ethanol and PBS. Antigens were retrieved by a 20-min incubation in hot EDTA buffer (10 mM Tris base, 1 mM EDTA, 0.05% Tween 20, pH = 9) in a steamer. After 20 minutes cooling at room temperature and 2 × 5 minutes washing with PBS, clusters were permeabilized with PBS-Tween 0,2% for five minutes. Thereafter, unspecific background was blocked with 5% FCS for 30 minutes at room temperature and primary antibody incubation for CHGA (Acris Antibodies AP15478PU-T, 1:300), CAIX (Novus Biologicals NB100-417, 1:200), CD56 (Biolegend MEM-188, 1:100) and Ki-67 (Ventana 790-4286, 1:3) diluted in 0,1% BSA took place in a humidity chamber at room temperature for one hour. Slides were washed 3 × 10 minutes in PBS-Tween 0,05% and corresponding Envision + HRP labelled polymer antibodies (Dako, Glostrup, Denmark) were applied to the slides for 30 minutes at room temperature in a humidity chamber. After 3 × 10 minutes washing steps with PBS-Tween 0,05%, visualization took place by adding DAB chromogen (Dako). Reaction was stopped by immersing into distilled water and cell nuclei were counterstained with hematoxylin (Dako). After eight seconds in PBS, and rinsing with distilled water, sections were mounted with Fluoromount-G (Southern Biotech, Birmingham, AL, USA). Aquisition of the microscopic images and quantitiative evaluation was done using the TissueFAXS System and HistoQuest software 4.0.4.159 (TissueGnostics, Vienna, Austria).

#### Scanning electron microscopy (SEM)

For SEM, samples were washed twice with PBS and fixed in Karnovsky’s fixative (2% paraformaldehyde, 2,5% glutaraldehyde in 0.1 M phosphate puffer pH 7.4; Morphisto®, Frankfurt am Main, Germany) and dehydrated in a graded ethanol series. Ethanol dehydration was followed by hexamethyldisilazane drying (HMDS, Sigma-Aldrich). Specimens were immersed in HMDS for 30 min and air dried. After complete evaporation of HMDS, samples were fixed to specimens mounts with double-faced adhesive tape, gold sputtered (Sputter Coater, SC502, Polaron, Fisons Instruments®, England) and then examined in a scanning electron microscope (JSM 6310, Jeol Ltd.®, Japan) at an acceleration voltage of 15 kV.

#### Cytotoxicity assay

Cytotoxicity was assessed using MTT assays and dilution steps of respective chemotherapeutics. In brief, 1 x 10^4^ cells in 100 µl medium as single cell suspension or tumorosphere were distributed to wells of 96-wells microtiter plates (TPP, Trasadingen Switzerland) and ten 2-fold dilutions of the chemotherapeutics were added in triplicate. Assays were at least performed in triplicate. Experimental observations suggest that chemotherapeutics reach only the first layers of tumor spheroids and we have shown that the SCLC CTC cells form a densely-closed surface layer of tumorospheres^41, 42^. Calculating CTC cell size and the surface area of the globular tumorospheres, 1 x 10^4^ suspension cells correspond to the same number of cells accessible at the surface of the spheroids when using 8 clusters of 0.2 mm diameter or 15 clusters of 0.15 mm diameter, respectively. The mean size of the clusters used for the cytotoxicity tests were determined and the number of clusters/well adjusted accordingly. The plates were incubated for four days under tissue culture conditions and viable cells detected using a modified MTT assay (EZ4U, Biomedica, Vienna, Austria). IC_50_ values were determined from dose-response curves using Origin 9.1 software (OriginLab, Northampton, MA, USA).

#### Cell cycle analysis

Cell cycle distributions were determined by propidium iodide (PI) staining and flow cytometry. 1 x 10^6^ cells per well were incubated in six-well plates for three days. Cells were harvested and fixed with 70% ethanol at −20 °C for 30 min and stained with 20 µg/ml propidium iodide (PI) and 5 µg/ml ribonuclease A in 0.05% NP40/PBS. Cells were analyzed by flow cytometry (Cytomics FC500) and MultiCycle AV software (Phoenix Flow Systems, San Diego, CA, USA).

#### Determination of sCAIX

Soluble CAIX was determined using an ELISA assay according to the manufacturer’s instructions (Human Carbonic Anhydrase IX Quantikine ELISA Kit; R&D Systems, Minneapolis, MN, USA)

#### Statistical analysis

Statistical significance was tested by t-tests and p < 0.05 regarded as significant difference.

## References

[1] Semenova EA, Nagel R, Berns A (2015). Origins, genetic landscape, and emerging therapies of small cell lung cancer. Genes Dev..

[2] Kalemkerian GP, Schneider BJ (2017). Advances in Small Cell Lung Cancer. Hematol. Oncol. Clin. North Am..

[3] Hamilton G, Rath B (2015). Smoking, inflammation and small cell lung cancer: recent developments. Wien Med. Wochenschr..

[4] Byers LA, Rudin CM (2015). Small cell lung cancer: where do we go from here?. Cancer.

[5] Rossi A (2016). Optimal drugs for second-line treatment of patients with small-cell lung cancer. Expert Opin. Pharmacother.

[6] Koinis F, Kotsakis A, Georgoulias V (2016). Small cell lung cancer (SCLC): no treatment advances in recent years. Transl. Lung Cancer Res..

[7] Santarpia M (2016). Targeted drugs in small-cell lung cancer. Transl. Lung Cancer Res..

[8] Hodgkinson CL (2014). Tumorigenicity and genetic profiling of circulating tumor cells in small-cell lung cancer. Nat. Med..

[9] Yu N, Zhou J, Cui F, Tang X (2015). Circulating tumor cells in lung cancer: detection methods and clinical applications. Lung.

[10] Celià-Terrassa T, Kang Y (2016). Distinctive properties of metastasis-initiating cells. Genes Dev..

[11] Hamilton G, Burghuber O, Zeillinger R (2015). Circulating tumor cells in small cell lung cancer: *ex vivo* expansion. Lung..

[12] Hamilton G, Hochmair M, Rath B, Klameth L, Zeillinger R (2016). Small cell lung cancer: Circulating tumor cells of extended stage patients express a mesenchymal-epithelial transition phenotype. Cell Adh. Migr..

[13] Dawood S, Austin L, Cristofanilli M (2014). Cancer stem cells: implications for cancer therapy. Oncology (Williston Park).

[14] Hamilton G, Rath B, Holzer S, Hochmair M (2016). Second-line therapy for small cell lung cancer: exploring the potential role of circulating tumor cells. Transl. Lung Cancer Res..

[15] Teicher BA (2014). Targets in small cell lung cancer. Biochem. Pharmacol..

[16] Rudin CM (2008). Randomized phase II Study of carboplatin and etoposide with or without the bcl-2 antisense oligonucleotide oblimersen for extensive-stage small-cell lung cancer: CALGB 30103. J. Clin. Oncol..

[17] Froehlich K (2016). Generation of Multicellular Breast Cancer Tumor Spheroids: Comparison of Different Protocols. J. Mammary Gland Biol. Neoplasia.

[18] Nath S, Devi GR (2016). Three-dimensional culture systems in cancer research: Focus on tumor spheroid model. Pharmacol. Ther..

[19] Weiswald LB, Bellet D, Dangles-Marie V (2015). Spherical cancer models in tumor biology. Neoplasia.

[20] Hirschhaeuser F (2010). Multicellular tumor spheroids: an underestimated tool is catching up again. J. Biotechnol..

[21] Sutherland RM (1988). Cell and environment interactions in tumor microregions: the multicell spheroid model. Science.

[22] Tannock IF, Lee CM, Tunggal JK, Cowan DS, Egorin MJ (2002). Limited penetration of anticancer drugs through tumor tissue: a potential cause of resistance of solid tumors to chemotherapy. Clin. Cancer Res..

[23] Desoize B, Jardillier J (2000). Multicellular resistance: a paradigm for clinical resistance?. Crit. Rev. Oncol. Hematol..

[24] St Croix B, Kerbel RS (1997). Cell adhesion and drug resistance in cancer. A major role for cell-cell adhesion in the regulation of intrinsic or acquired resistance. Curr. Opin. Oncol..

[25] Mellor HR, Ferguson DJ, Callaghan R (2005). A model of quiescent tumour microregions for evaluating multicellular resistance to chemotherapeutic drugs. Br. J. Cancer.

[26] Däster, S. *et al*. Induction of hypoxia and necrosis in multicellular tumor spheroids is associated with resistance to chemotherapy treatment. *Oncotarget* (2016). 10.18632/oncotarget.13857 PMC5352092 27965457

[27] Laurent J (2013). Multicellular tumor spheroid models to explore cell cycle checkpoints in 3D. BMC Cancer.

[28] Olive PL (2001). Carbonic anhydrase 9 as an endogenous marker for hypoxic cells in cervical cancer. Cancer Res..

[29] Ilie M (2010). High levels of carbonic anhydrase IX in tumour tissue and plasma are biomarkers of poor prognostic in patients with non-small cell lung cancer. Br. J. Cancer.

[30] Zschenker, O, Streichert, T., Hehlgans, S. & Cordes, N. Genome-wide gene expression analysis in cancer cells reveals 3D growth to affect ECM and processes associated with cell adhesion but not DNA repair. *PLoS One* **7** (2012). 10.1371/journal.pone.0034279 PMC3324525 22509286

[31] Mehta G, Hsiao AY, Ingram M, Luker GD, Takayama S (2012). Opportunities and challenges for use of tumor spheroids as models to test drug delivery and efficacy. J. Control Release.

[32] Pease, J. C., Brewer, M., Tirnauer, J.S. Spontaneous spheroid budding from monolayers: a potential contribution to ovarian cancer dissemination. *Biol. Open* **1**, 622–628 (2012). 10.1242/bio.2012653 PMC3507299 23213456

[33] Chitcholtan K, Sykes PH, Evans JJ (2012). The resistance of intracellular mediators to doxorubicin and cisplatin are distinct in 3D and 2D endometrial cancer. J. Transl. Med..

[34] Moreno L, Pearson AD (2013). How can attrition rates be reduced in cancer drug discovery? Expert Opin. Drug Discov..

[35] Jardim DL, Groves ES, Breitfeld PP, Kurzrock R (2017). Factors associated with failure of oncology drugs in late-stage clinical development: A systematic review. Cancer Treat. Rev..

[36] Pampaloni F, Reynaud EG, Stelzer EH (2007). The third dimension bridges the gap between cell culture and live tissue. Nat. Rev. Mol. Cell Biol..

[37] Hoffmann OI (2015). Impact of the spheroid model complexity on drug response. J. Biotechnol..

[38] Shield K, Ackland ML, Ahmed N, Rice GE (2009). Multicellular spheroids in ovarian cancer metastases: Biology and pathology. Gynecol Oncol..

[39] Sodek KL, Murphy KJ, Brown TJ, Ringuette MJ (2012). Cell-cell and cell-matrix dynamics in intraperitoneal cancer metastasis. Cancer Metastasis Rev..

[40] Gasch C, Ffrench B, O’Leary JJ, Gallagher MF (2017). Catching moving targets: cancer stem cell hierarchies, therapy-resistance & considerations for clinical intervention. Mol. Cancer.

[41] Erlichman C, Vidgen D (1984). Cytotoxicity of adriamycin in MGH-U1 cells grown as monolayer cultures, spheroids, and xenografts in immune-deprived mice. Cancer Res..

[42] Hamilton G, Moser D, Hochmaier M (2016). Metastasis: Circulating Tumor Cells in Small Cell Lung Cancer. Trends Cancer.

